# Microwave-assisted synthesis of some hybrid molecules containing penicillanic acid or cephalosporanic acid moieties and investigation of their biological activities

**DOI:** 10.1007/s00044-013-0898-4

**Published:** 2013-12-29

**Authors:** Serap Basoglu, Serdar Ulker, Sengul Alpay-Karaoglu, Neslihan Demirbas

**Affiliations:** 1 Department of Chemistry, Faculty of Sciences, Karadeniz Technical University, 61080 Trabzon, Turkey; 2 Department of Biology, Recep Tayyip Erdogan University, 53100 Rize, Turkey

**Keywords:** Piperazine, 1,3-Oxa(thia)zole, 5-Oxo-1,3-oxazolidine, 1,2,4-Triazole, 7-Aminocephalosporanic acid, 6-Aminopenicillanic acid, Biological activity

## Abstract

Ethyl 4-amino-2-fluorophenylpiperazin-1-carboxylates containing a 1,3-oxazol(idin)e, 5-thioxo-1,2,4-triazole, 1,3,4-thiadiazole, 5-thioxo-1,3,4-oxadiazole, or 1,3-thiazole nucleus were obtained starting from ethyl piperazine-1-carboxylate (**1**) by several steps. The treatment of amine, **3** or hydrazide, **9** with several aromatic aldehydes generated the corresponding arylmethyleneamino (**3a**–**f**) or arylidenehydrazino (**12a**–**c**) compounds. The Mannich reaction between the 1,2,4-triazole or 1,3,4-oxadiazole compounds and 7-aca produced cephalosporanic acid derivatives. Penicillanic acid derivatives were obtained when 6-apa was used in the Mannich reactions. The synthesized compounds were screened for their antimicrobial, antilipase, and antiurease activities. Some of them were found to possess good-moderate antimicrobial activity against the test microorganisms. Two compounds exhibited antiurease activity, and four of them displayed antilipase activity.

## Introduction

The limitations of the existing antibacterial drugs caused by various reasons including drug resistance, the serious side effects, and/or lack of efficacy made infectious diseases a vicious cycle. In addition, the treatment of resistant strains requires a prolonged therapy containing the use of more toxic drugs and increases the financial burden. The rising prevalence of multi-drug resistant bacteria continues to serve medicinal chemists to search and discove novel antimicrobial agents effective against pathogenic microorganisms resistant to current treatment.

Among the strategies addressed to the synthesis of compounds possessing antimicrobial activity, the syntheses of hybrid molecules incorporating different heterocyclic moieties have been attracting widespread attention (Mallikarjuna *et al*., 2009).

A number of N-containing heterocyclic compounds constitute important building blocks in organic and medicinal chemistry. For example, triazoles have been shown to possess a number of desirable activities in the context of medicinal chemistry. Ribavirin (antiviral), rizatriptan (antimigraine), alprazolam (psychotropic), fluconazole, and itraconazole (antifungal) are the best examples for potent drugs possessing triazole nucleus (Holla *et al*., 2006; Walczak *et al*., 2004; Jones *et al*., 1965; Ashok *et al*., 2007). Tazobactam, a *β*-lactamase inhibitor is the other best known example of triazole containing structures with the broad spectrum antibiotic piperacillin (Kategaonkar *et al*., 2010).

Substituted piperazines constitute another class of important pharmacophores, which are found in many marketed drugs, such as the HIV protease inhibitor, Crixivan (Chaudhary *et al*., 2006). Ciprofloxacin, norfloxacin, pefloxacine, ofloxacin, and enoxacin are fluoroquinolone class antibacterial drugs characterized by having a piperazine moiety at C-7 of quinolone skeleton, and they have been used for the treatment of bacterial infections (Foroumadi *et al*., 2005).

The compounds having a thiazolidinone nucleus are of interest due to their broad spectrum of biological activities such as bactericidal, fungicidal, antimicrobial, antiproliferative, antiviral, anticonvulsant, anticancer, and anti-inflammatory activities (Vicini *et al*., 2008; Wang *et al*., 2011; Lv *et al*., 2010; Metwally *et al*., 2010; Balzarini *et al*., 2009; Havrylyuk *et al*., 2009; Subtelna *et al*., 2010; Mushtaque *et al*., 2012).

Mannich bases, which are known to be physiologically reactive since their basic function rendering the molecule soluble in aqueous solvents when it is transformed into aminium salt, have been reported as potential biological agents (Karthikeyan *et al*., 2006). *N*-Mannich bases have been used successfully to obtain prodrugs of amine as well as amide-containing drugs (Zhao *et al*., 2009). Some Mannich bases derived from 1,2,4-triazole nucleus have been reported to possess protozocidal and antibacterial activity (Ashok *et al*., 2007; Almajan *et al*., 2009; Bayrak *et al*., 2009, 2010; Demirbas *et al*., 2009; Bektas *et al*., 2010; Patole *et al*., 2006).

Schiff bases have gained importance in medicinal and pharmaceutical fields due to their most versatile properties as organic synthetic intermediates and also possessing a broad range of biological activities, such as antituberculosis, anticancer, analgesic and anti-inflammatory, anticonvulsant, antibacterial, and antifungal activities (Patole *et al*., 2006, Hearn and Cynamon, 2004; Ren *et al*., 2002; Demirbas *et al*., 2002; Lohray *et al*., 2006).

We envisage that hybrid compound incorporating a 4-(2-fluorophenylene)-piperazine core with several heterocyclic moieties responsible for biological activity in a single molecular frame could lead to the novel potent antimicrobial and antiurease agents. Highly substituted piperazines can be expected to increase antimicrobial activity probably by enhancing lipophilicity of molecule.

In continuation of our research program on the synthesis of hybrid molecules containing various heterocyclic moieties, we planned the synthesis of 4-(2-fluorophenyl)piperazine derivatives along with their antimicrobial and antiurease activities.

## Results and discussion

**Scheme 1:**
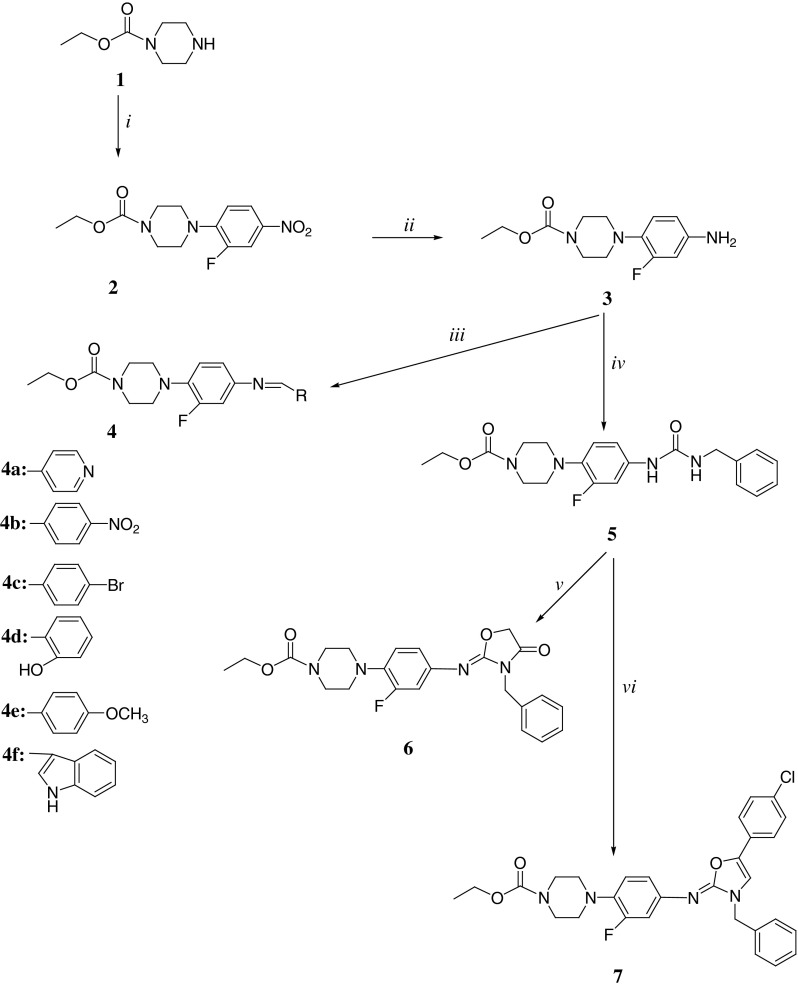
*i* 3,4-Difluoronitrobenzene in ethanol, reflux for 6 h. *ii* Pd–C, hydrazine hydrate in *n*-butanol, reflux for 7 h. *iii* Indole-3-carboxaldehyde in absolute ethanol, irradiation by MW at 150 W, 110 °C for 30 min. *iv* Benzylisothiocyanate in absolute ethanol, reflux for 10 h. *v* Ethyl bromoacetate in absolute ethanol, dried sodium acetate, reflux for 13 h. *vi* 4-Chlorophenacylbromide in absolute ethanol, dried sodium acetate, reflux for 11 h

**Scheme 2:**
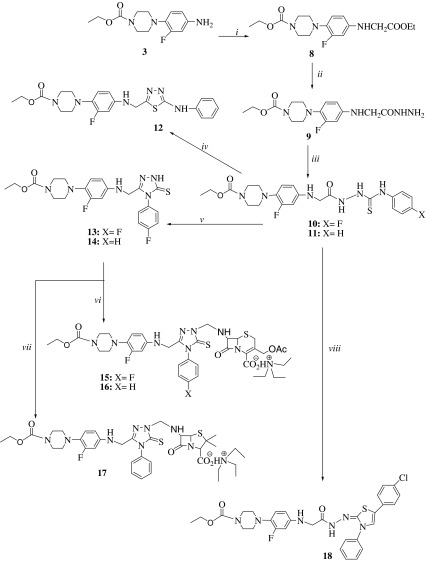
*i* Ethyl bromoacetate, Et_3_N, THF, rt for 14 h. *ii* Hydrazine hydrate in ethanol, reflux for 14 h. *iii* 4-Fluorophenylisothiocyanate or phenylisothiocyanate in absolute ethanol, reflux for 10 h. *iv* H_2_SO_4_, rt for 2 h. *v* NaOH in water, reflux for 3 h. *vi* 7-Aca, HCHO, Et_3_N in THF, rt, for 4 h. *vii* 6-Apa, HCHO, Et_3_N in THF, rt, for 4 h. *vii* 4-Chlorophenacylbromide in absolute ethanol, dried sodium acetate, reflux for 12 h

**Scheme 3:**
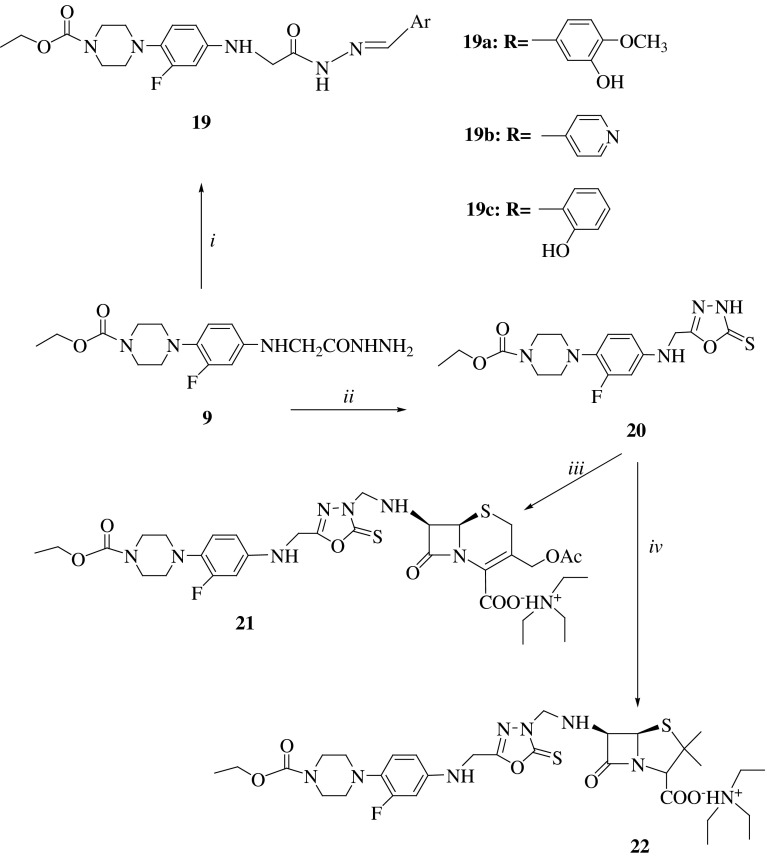
*i* 3-Hydroxy-4-phenoxybenzaldehyde, pyridine-4-carbaldehyde, 2-hydroxybenzaldehyde in absolute ethanol, irradiation by MW at 200 W, 140 °C for 30 min. *ii* CS_2_ and KOH in ethanol, reflux for 13 h. *iii* 7-Aca, HCHO, Et_3_N in THF, rt, for 4 h. *iv* 6-Apa, HCHO, Et_3_N in THF, rt, for 4 h

The main aim of the present study is the synthesis and antimicrobial activity evaluation of new piperazine derivatives incorporating several heterocyclic moieties including 1,3-oxadiazole, 1,2,4-triazole, 1,3-oxa(thia)zole, penicillanic acid, and/or cephalosporanic acid. Synthesis of the intermediate and target compounds was performed according to the reactions outlined in Schemes 1, 2, and 3. The starting compound ethyl 1-piperazinecarboxylate (**1**) was provided commercially.

Ethyl 4-(4-amino-2-fluorophenyl)piperazine-1-carboxylate (**3**), that was obtained starting from compound **1** by two steps, was converted to the corresponding arylmethylenamino derivatives (**4a**–**f**) by the treatment with several aromatic aldehydes. In the FT-IR and ^1^H NMR spectra of these compounds, no signal pointing the –NH_2_ group was seen. Instead, additional signals derived from aldehyde moiety were recorded at the related chemical shift values in the ^1^H NMR spectra.

The cyclocondensation of compound **5**, that was obtained from the reaction of **4** with benzylisocyanate, with ethyl bromoacetate or 4-chlorophenacyl bromide produced the corresponding hybrid molecules incorporating a 4-oxo-1,3-oxazolidine (**6**) or 4-chlorophenyl)-1,3-oxazole (**7**) nucleus in the 2-fluorophenylpiperazine-1-carboxylate skeleton. The ^1^H and ^13^C NMR spectra of compound **7** exhibited additional signals at aromatic region originated from 4-chlorophenyl nucleus as a result of condensation. Moreover, the elemental analyses and mass spectral data of derivatives **6** and **7** were compatible with the suggested structures.

The treatment of compound **3** with ethyl bromoacetate at room temperature in the presence of triethylamine resulted in the formation of compound **8**. When compound **8** was converted to the corresponding hydrazide (**9**) by refluxing with hydrazine hydrate, the signals originated from ester function was disappeared in the ^1^H and ^13^C NMR spectra. Instead, new signals due to –NHNH_2_ protons were seen at 5.93 and 9.09 ppm. Meanwhile, the stretching frequency band of this group was recorded at 3,313 cm^−1^ as a wide signal characteristic for the hydrazide structure. Compounds **6** and **7** gave mass fragmentation confirming the proposed structures.

The synthesis of compounds **10** and **11** was carried out by the treatment of compound **7** with the corresponding isothiocanates. These compounds displayed spectroscopic data and elemental analysis results consistent with the assigned structures.

The intramolecular cyclization of compound **10** generated the corresponding 1,3,4-thiazole compound (**12**) in acidic media. On the other hand, the basic treatment of compounds **10** and **11** caused to the cyclization of the (arylamino)carbonothioylhydrazino side change leading to the formation of 5-thioxo-4,5-dihydro-1*H*-1,2,4-triazol derivatives (**13** and 1**4**). With the conversion of compounds **10** and **11** to compounds **12**–**14**, two of NH signals were disappeared in the ^1^H NMR spectra. It is well-known that type of compounds can stay in thioxo or mercapto tautomeric form. In the present study, compounds **13** and **14** are present predominately in the thioxo form as it was shown by the C=S band at 1,244–1,250 cm^−1^ in the FT-IR spectra of these compounds. Furthermore, the ^1^H NMR spectra of compounds **13** and **14** revealed clearly the absence of the signal originated from SH proton, instead of that, two signals due to NH proton on 1,2,4-triazol ring was recorded at 10.45 (for **13**) or 11.27 (for **14**), that is characteristic for 4,5-dihydro-1*H*-1,2,4-triazoles.

The synthesis of Mannich bases (**15**–**17**) was performed by the reaction of compounds **13** and **14** with 6-aminopenicillanic acid, 6-apa (for **17**) or 7-aminocephalosporanic acid, 7-aca (for **15** and **16**) in tetrahydrofuran at room temperature in the presence of triethylamine and formaldehyde. The occurrence of the alkylaminomethylation was provided by the disappearance of signal for the proton at the *N*-1 nitrogen of the 1,2,4-triazole ring. Moreover, in ^1^H and ^13^C NMR spectra, additional signal corresponding to the 6-apa or 7-aca-ammonium salt was recorded at the related chemical shift value.

The conversion of arylcarbonothioylhydrazino side change to 4-chlorophenyl-3-phenyl-1,3-thiazole ring (**18**) was accomplished with the treatment of 4-chlorophenacyl bromide. This compound was characterized by spectroscopic techniques including ^1^H NMR, ^13^C NMR, FT-IR, EI-MS, and elemental analysis.

The synthesis of ethyl arylidenehydrazino-piperazine-1-carboxylate derivatives (**19a**–**c**) was performed by microwave irradiation of compound **9** with several aromatic aldehydes namely 3-hydroxy-4-methoxybenzaldehyde, pyridine-4-carbaldehyde, and 2-hydroxybenzaldehyde. In the FT-IR spectra of these arylidenehydrazino compounds, absorption bands characteristic for NH groups were visible in the ranges of 3,357–3,181 cm^−1^. Another piece of evidence for condensation was the appearance of a signal as singlet integrating for one proton in the ^1^H NMR spectra, which corresponds to the N=CH proton of azomethyne group. Moreover, these compounds gave mass fragmentation and elemental analysis confirming the proposed structures.

Ethyl 4-(2-fluoro-4-{[(5-thioxo-4,5-dihydro-1,3,4-oxadiazol-2-yl)methyl]amino} phenyl)piperazine-1-carboxylate (**20**) was prepared from the reaction of compound **9** with CS_2_ in the basic media. The attempts for aminoalkylations of compound (**20**) by Mannich reaction allowed the isolation of the corresponding products (**21** and **22**) after 4 (for **21**) or 6 h (for **22**) at room temperature. This idea originated from the intent to introduce the penicillanic acid or cephalosporanic acid nucleus to (piperazin-1-yl)-2-thioxo-1,3,4-oxadiazole skeleton. As different from **20**, the NMR spectra of the obtained Mannich bases (**21** and **22**) displayed additional signals derived from penicillanic- or cephalosporanic-acid moiety and –CH_2_—linkage at the related shift and integral values as D_2_O nonexchangeable signals.

Among the synthesized compounds ethyl 4-(2-fluoro-4-nitrophenyl)piperazine-1-carboxylate (**2**) exhibited activity on *Bacillus cereus* (Bc), that is Gram positive spore bacillus. With the reduction of nitro group of **2** to amine (compound **3**), additional activities towards *Staphylococcus aureus* (Sa), that is Gram positive coccus, *Candida albicans* (Ca), and *Saccharomyces cerevisiae* (Sc), which are yeast like fungi. For the imine compounds (**4a**–**f**), the highest activity was observed against *Mycobacterium smegmatis* (Ms) that is an atypical tuberculosis factor leading mortality, with the inhibition zone varying between 10 and 25 mm. The compounds containing 1,2,4-triazole and cephalosporanic- or penicillanic-acid moiety (compounds **15**–**17**) displayed good-moderate activity on some of the test microorganisms. The highest activity was observed for compound **17** on Bc with the inhibition zone of 16 mm. This result is better than standard drug ampicillin. Other compounds containing penicillanic acid or cephalosporanic acid core (**21** and **22**) displayed good-moderate activity against the test microorganisms.

The synthesized compounds were assayed for their in vitro urease inhibitory activity against Jack bean urease. Two of those compounds showed perfect urease inhibition. No inhibitory effect was detected for other compounds. Thiourea with IC_50_ value 54.56 ± 4.17 μg mL^−1^ was used as standard inhibitor. Among tested compounds, compound **15** was found to be the best inhibitory effect against urease with an IC_50_ value of 4.67 ± 0.53 μg mL^−1^. At the various final concentrations the compound **15** showed more inhibitory effect than standard urease inhibitor thiourea. Also, compound **17** has the highest inhibitory activity than thiourea. These compounds might be considered as potential antibiotics to treat infections.

All compounds were evaluated with regard to pancreatic lipase activity and compounds **12**, **13**, **14**, and **15**, which are 1,3,4-thiadizole or 1,2,4-triazole derivatives including also 4-fluorophenylpiperazine nucleus, showed moderate anti-lipase activities at final concentration of 6.25 μg mL^−1^. No inhibitory effect was detected for other compounds. Orlistat, known pancreatic lipase inhibitor used as anti-obesity drug, showed inhibitory effect by 99 % at the same concentration.

## Conclusion

This study reports microwave-assisted synthesis of some new hybrid molecules containing penicillanic acid or cephalosporanic acid moieties with some other pharmacophore heterocycles in a single structure. Hence herein we combined all these potential chemotherapeutic units, namely 1,2,4-triazole, 1,3-thiazole, 1,3-oxazole, 1,3,4-oxadiazole, piperazine, penicillanic acid, cephalosporanic acid moieties. The antimicrobial, antiurease, and antilipase screening studies were also performed in the study.

Among the synthesized compounds, the compounds containing 1,2,4-triazole and cephalosporanic- or penicillanic-acid moiety (**15**–**17**) displayed good-moderate activity on some of the test microorganisms. The highest activity was observed for compound **17** on Bc with the inhibition zone of 16 mm. This result is better than standard drug ampicillin. Moreover, compounds **15** and **17** exhibited an inhibitory effect against urease. Other compounds containing penicillanic acid or cephalosporanic acid core (**21** and **22**) displayed good-moderate activity against the test microorganisms. Furthermore, compounds **12**, **13**, **14**, and **15**, which are 1,3,4-thiadizole or 1,2,4-triazole derivatives including also 4-fluorophenylpiperazine nucleus, showed moderate anti-lipase activities at final concentration of 6.25 μg mL^−1^.

## Experimental

### Chemistry

#### General information for chemicals

All the chemicals were purchased from Fluka Chemie AG Buchs (Switzerland) and used without further purification. Melting points of the synthesized compounds were determined in open capillaries on a Büchi B-540 melting point apparatus and are uncorrected. Reactions were monitored by thin-layer chromatography (TLC) on silica gel 60 F254 aluminum sheets. The mobile phase was ethyl acetate:diethyl ether, 1:1, and detection was made using UV light. FT-IR spectra were recorded as potassium bromide pellets using a Perkin Elmer 1600 series FT-IR spectrometer. ^1^H NMR and ^13^C NMR spectra were registered in DMSO-*d*_6_ on a BRUKER AVANCE II 400 MHz NMR Spectrometer (400.13 MHz for ^1^H and 100.62 MHz for ^13^C). The chemical shifts are given in ppm relative to Me_4_Si as an internal reference, *J* values are given in Hz. The elemental analysis was performed on a Costech Elemental Combustion System CHNS–O elemental analyzer. All the compounds gave C, H, and N analysis within ±0.4 % of the theoretical values. The mass spectra were obtained on a Quattro LC–MS (70 eV) instrument.

##### Ethyl 4-(2-fluoro-4-nitrophenyl)piperazine-1-carboxylate (**2**)

The solution of 3,4-difluoronitrobenzene (10 mmol) in excess amount of ethyl 1-piperazinecarboxylate (40 mmol) was allowed to reflux for 6 h (the progress of the reaction was monitored by TLC). Then, the mixture was poured into ice-water. The precipitated product was filtered off and recrystallized from ethanol. Yield 97 %, m.p: 90–93 °C. FT-IR (KBr, *ν*, cm^−1^): 3099 (ar–CH), 1509, and 1354 (NO_2_). Elemental analysis for C_13_H_16_FN_3_O_4_ calculated (%): C, 52.52; H, 5.42; N, 14.13. Found (%): C, 52.64; H, 5.70; N, 14.00. ^1^H NMR (DMSO-*d*_6_, *δ* ppm): 1.19 (t, 3H, CH_3_, *J* = 7.0 Hz), 3.26 (s, 4H, 2CH_2_), 3.51 (s, 4H, 2CH_2_), 4.06 (q, 2H, CH_2_, *J* = 6.6 Hz), 7.16 (t, 1H, arH, *J* = 7.8 Hz), 8.00 (d, 2H, arH, *J* = 7.8 Hz). ^13^C NMR (DMSO-*d*_6_, *δ* ppm): 11.47 (CH_3_), 40.46 (2CH_2_), 45.81 (2CH_2_), 57.92 (CH_2_), arC: [105.00 (CH), 109.09 (d, CH, *J*_C–F_ = 26.0 Hz), 116.54 (d, CH, *J*_C–F_ = 154.0 Hz), 136.43 (C), 142.01 (C), 146.05 (C)], 151.46 (C=O). MS *m*/*z* (%): 301.29 (32), 167.01 (18), 159.03 (19), 148.96 (100), 113.05 (34).

##### Ethyl 4-(4-amino-2-fluorophenyl)piperazine-1-carboxylate (**3**)

Pd–C (5 mmol) catalyst was added to the solution of compound (**2**) (10 mmol) in *n*-butanol, and the mixture was refluxed in the presence of hydrazine hydrate (50 mmol) for 7 h. The progress of the reaction was monitored by TLC. Then, the catalyst was separated by filtration and the solvent was evaporated under reduced pressure. The solid obtained was recrystallized from ethanol. Yield 65 %. M.p: 116–119 °C. FT-IR (KBr, *ν*, cm^−1^): 3,423 and 3,341 (NH_2_), 1682 (C=O). Elemental analysis for C_13_H_18_FN_3_O_2_ calculated (%): C, 58.41; H, 6.79; N, 15.72. Found (%): C, 58.31; H, 6.87; N, 15.78. ^1^H NMR (DMSO-*d*_6_, *δ* ppm): 1.18 (t, 3H, CH_3_, *J* = 7.0 Hz), 2.76 (s, 4H, 2CH_2_), 3.45 (s, 4H, 2CH_2_), 4.04 (q, 2H, CH_2_, *J* = 7.4 Hz), 5.03 (s, 2H, NH_2_), 6.33 (d, 2H, arH, *J* = 12.4 Hz), 6.76 (t, 1H, arH, *J* = 9.0 Hz). ^13^C NMR (DMSO-*d*_6_, *δ* ppm): 14.53 (CH_3_), 43.56 (2CH_2_), 51.07 (2CH_2_), 60.75 (CH_2_), arC: [101.66 (d, CH, *J*_C–F_ = 23.0 Hz), 109.39 (CH), 120.92 (d, CH, *J*_C–F_ = 4.05 Hz), 128.70 (d, C, *J*_C–F_ = 9.5 Hz), 145.72 (d, C, *J* = 10.6 Hz), 154.18 (d, C, *J*_C–F_ = 34.5 Hz)], 158.65 (C=O). MS *m*/*z* (%): 268.10 ([M+1]^+^,100).

##### Ethyl 4-(2-fluoro-4-{[pyridin-4-ylmethylene]amino}phenyl)piperazine-1-carboxylate (**4a**)

Indole-3-carboxaldehyde (10 mmol) was added to the solution of compound **3** (10 mmol) in absolute ethanol and the reaction mixture was irradiated by microwave at 150 W and 110 °C for 30 min. After removing in the solvent under reduced pressure, an oily product obtained. This was recrystallized from butyl acetate and diethyl ether (1:2). Yield: 81 %, M.p: 162–163 °C. FT-IR (KBr, *ν*, cm^−1^): 1686 (C=O), 1508 (C=N), 1224 (C–O). Elemental analysis for C_19_H_21_FN_4_O_2_ calculated (%): C, 64.03; H, 5.94; N, 15.72. Found (%): C, 64.18; H, 6.14; N, 15.78. ^1^H NMR (DMSO-*d*_6_, *δ* ppm): 1.19 (t, 3H, CH_3_, *J* = 6.6 Hz), 3.00 (s, 4H, 2CH_2_), 3.51 (s, 4H, 2CH_2_ + H_2_O), 4.04–4.11 (m, 2H, CH_2_), 7.04–7.34 (m, 3H, arH), 7.80 (d, 2H, arH, *J* = 4.2 Hz), 8.71 (s, 3H, arH + N=CH). ^13^C NMR (DMSO-*d*_6_, *δ* ppm): 15.26 (CH_3_), 44.01 (CH_2_), 50.69 (CH_2_), 51.83 (2CH_2_), 61.57 (CH_2_), arC: [102.18 (CH), 109.63 (d, CH, *J*_C–F_ = 21.0 Hz), 120.05 (d, CH, *J*_C–F_ = 31.5 Hz), 121.37 (C), 122.77 (2CH), 139.48 (d, C, *J*_C–F_ = 9.0 Hz), 144.37 (d, C, *J*_C–F_ = 120.0 Hz), 151.14 (2CH), 154.23 (d, C, *J*_C–F_ = 103.2 Hz)], 158.09 (N=CH), 158.90 (C=O). MS *m*/*z* (%): 357.11 ([M+1]^+^, 64), 302.10 (100), 342.24 (80).

##### Ethyl 4-(2-fluoro-4-{[(4-nitrophenyl)methylene]amino}phenyl)piperazine-1-carboxylate (**4b**)

The mixture of compound **3** (10 mmol) and 4-nitrobenzaldehyde (10 mmol) in absolute ethanol was irradiated by microwave at 150 W and 110 °C for 10 min. The solid obtained was recrystallized from ethyl acetate:petroleum ether (1:2). Yield: 58 %, M.p: 164–166 °C. FT-IR (KBr, *ν*, cm^−1^): 3074 (ar–CH), 1696 (C=O), 1510, and 1341 (NO_2_), 1433 (C=N), 1215 (C–O). Elemental analysis for C_20_H_21_FN_4_O_4_ calculated (%): C, 59.99; H, 5.29; N, 13.99. Found (%): C, 60.12; H, 5.45; N, 14.19. ^1^H NMR (DMSO-*d*_6_, *δ* ppm): 1.19 (brs, 3H, CH_3_), 3.10 (s, 4H, 2CH_2_), 3.51 (s, 4H, 2CH_2_), 4.04–4.17 (m, 2H, CH_2_), 7.09–7.37 (m, 3H, arH), 8.14 (d, 2H, arH, *J* = 7.8 Hz), 8.35 (d, 2H, arH, *J* = 7.8 Hz), 8.84 (s, 1H, N=CH). ^13^C NMR (DMSO-*d*_6_, *δ* ppm): 15.16 (CH_3_), 43.39 (CH_2_), 50.64 (CH_2_), 55.44 (2CH_2_), 61.57 (CH_2_), arC: [110.57 (d, CH, *J*_C–F_ = 32.7 Hz), 117.30 (CH), 120.24 (d, CH, *J*_C–F_ = 41.0 Hz), 123.62 (d, C, *J*_C–F_ = 42.5 Hz), 124.76 (2CH), 130.18 (2CH), 139.53 (C), 142.26 (C), 146.09 (d, C, *J*_C–F_ = 51.0 Hz), 150.35 (d, C, *J*_C–F_ = 96.0 Hz)], 153.46 (C=O), 160.32 (N=CH).

##### Ethyl 4-(4-{[(4-bromophenyl)methylene]amino}-2-fluorophenyl)piperazine-1-carboxylate (**4c**)

The mixture of compound **3** (10 mmol) and 4-bromobenzaldehyde (10 mmol) in absolute ethanol was irradiated at 150 W and 150 °C for 30 min. The yellow solid obtained was recrystallized ethanol. Yield: 84 %, M.p: 124–126 °C. FT-IR (KBr, *ν*, cm^−1^): 3053 (ar–CH), 1671 (C=O), 1434 (C=N), 1210 (C–O). Elemental analysis for C_20_H_21_BrFN_3_O_2_ calculated (%): C, 55.31; H, 4.87; N, 9.68. Found (%): C, 55.71; H, 4.90; N, 9.79. ^1^H NMR (DMSO-*d*_6_, *δ* ppm): 1.19 (t, 3H, CH_3_, *J* = 7.0 Hz), 2.98 (s, 4H, 2CH_2_), 3.51 (s, 4H, 2CH_2_), 4.05 (q, 2H, CH_2_, *J* = 7.0 Hz), 6.93–7.27 (m, 3H, arH), 7.71 (d, 2H, arH, *J* = 7.8 Hz), 7.84 (d, 2H, arH, *J* = 8.2 Hz), 8.65 (s, 1H, N=CH). ^13^C NMR (DMSO-*d*_6_, *δ* ppm): 15.26 (CH_3_), 41.40 (CH_2_), 44.04 (CH_2_), 50.78 (2CH_2_), 61.56 (CH_2_), arC: [105.00 (CH), 109.44 (d, CH, *J*_C–F_ = 22.5 Hz), 119.80 (d, CH, *J*_C–F_ = 58.2 Hz), 125.61 (C), 131.05 (2CH), 132.57 (2CH), 135.83 (C), 138.83 (d, C, *J*_C–F_ = 8.75 Hz), 146.26 (d, C, *J*_C–F_ = 8.5 Hz), 153.39 (C)], 155.27 (C=O), 159.44 (N=CH).

##### Ethyl 4-{2-fluoro-4-[(2-hydroxybenzylidene)amino]phenyl}piperazine-1-carboxylate (**4d**)

The solution of compound **3** (10 mmol) in absolute ethanol was refluxed with 2-hydroxybenzaldehyde (10 mmol) for 7 h. On cooling the reaction content to room temperature, a solid appeared. This crude product was filtered off and recrystallized from acetone. Yield: 83 %. M.p: 136–137 °C. FT-IR (KBr, *ν*, cm^−1^):1697 (C=O), 1510 (C=N), 1225 (C–O). Elemental analysis for C_20_H_22_FN_3_O_3_ calculated (%): C, 64.68; H, 5.97; N, 11.31. Found (%): C: 64.31; H: 5.78; N: 11.48. ^1^H NMR (DMSO-*d*_6_, *δ* ppm): 1.21 (brs, 3H, CH_3_), 3.00 (s, 4H, 2CH_2_), 3.52 (s, 4H, 2CH_2_), 4.06 (brs, 2H, CH_2_), 6.97–7.59 (m, 7H, arH), 8.95 (s, 1H, N=CH), 13.02 (s, 1H, OH). ^13^C NMR (DMSO-*d*_6_, *δ* ppm): 15.26 (CH_3_), 44.40 (2CH_2_), 50.66 (2CH_2_), 61.59 (CH_2_), arC: [109.50 (d, CH, *J*_C–F_ = 22.0 Hz), 117.24 (2CH), 119.33 (CH), 119.87 (C), 120.22 (d, CH, *J*_C–F_ = 28.5 Hz), 133.18 (CH), 133.86 (CH), 139.28 (d, C, *J*_C–F_ = 9.0 Hz), 143.26 (d, C, *J*_C–F_ = 8.5 Hz), 153.32 (C), 156.74 (d, C, *J*_C–F_ = 145.5 Hz)], 160.82 (C=O), 163.17 (N=CH).

##### Ethyl 4-(2-fluoro-4-{[(4-methoxyphenyl)methylene]amino}phenyl)piperazine-1-carboxylate (**4e**)

The solution of compound **3** (10 mmol) in absolute ethanol was refluxed with 4-methoxybenzaldehyde (10 mmol) for 7 h. On cooling the reaction content to room temperature, a solid appeared. This crude product was filtered off and recrystallized from ethanol. Yield: 42 %. M.p: 122–124 °C. FT-IR (KBr, *ν*, cm^−1^): 1688 (C=O), 1509 (C=N), 1225 (C–O). Elemental analysis for C_21_H_24_FN_3_O_3_ calculated (%): C, 65.44; H, 6.28; N, 10.90. Found (%): C, 65.56; H, 6.52; N, 11.12. ^1^H NMR (DMSO-*d*_6_, *δ* ppm): 1.19 (t, 3H, CH_3_, *J* = 6.6 Hz), 2.96 (s, 4H, 2CH_2_), 3.49 (s, 4H, 2CH_2_), 3.82 (s, 3H, O–CH_3_), 4.06 (q, 2H, CH_2_, *J* = 6.8 Hz), 6.71–6.78 (m, 1H, arH), 7.04–7.22 (m, 5H, arH), 7.86 (d, 1H, arH, *J* = 8.2 Hz), 8.58 (s, 1H, N=CH). ^13^C NMR (DMSO-*d*_6_, *δ* ppm): 15.27 (CH_3_), 44.13 (CH_2_), 50.85 (CH_2_), 51.35 (2CH_2_), 56.10 (O–CH_3_) 61.53 (CH_2_), arC: [109.73 (d, CH, *J*_C–F_ = 38.9 Hz), 114.98 (2CH), 118.72 (CH), 121.90 (d, CH, *J*_C–F_ = 66.3 Hz), 129.12 (C), 131.24 (2CH), 132.53 (C), 138.35 (d, C, *J*_C–F_ = 21.0 Hz), 147.24 (C), 154.40 (d, C, *J*_C–F_ = 94.5 Hz), 160.10 (N=CH), 162.24 (C=O).

##### Ethyl 4-(2-fluoro-4-{[1*H*-indol-3-ylmethylene]amino}phenyl)piperazine-1-carboxylate (**4f**)

The solution of compound **3** (10 mmol) in absolute ethanol was refluxed with indol-3-carbaldehyde (10 mmol) for 6 h. On cooling the reaction content to room temperature, a solid appeared. This crude product was filtered off and recrystallized from acetone. Yield: 82 %. M.p: 184–186 °C. FT-IR (KBr, *ν*, cm^−1^): 3484 (NH), 1678 (C=O), 1439 (C=N), 1220 (C–O). Elemental analysis for C_22_H_23_FN_4_O_2_ calculated (%): C, 66.99; H, 5.88; N, 14.20. Found (%): C, 66.76; H, 6.02; N, 14.01. ^1^H NMR (DMSO-*d*_6_, *δ* ppm): 1.20 (brs, 3H, CH_3_), 3.01 (s, 4H, 2CH_2_), 3.53 (s, 4H, 2CH_2_), 4.06 (brs, 2H, CH_2_), 7.29 (brs, 5H, arH), 8.08 (s, 1H, arH), 8.38 (s, 2H, arH), 9.06 (s, 1H, N=CH), 9.29 (s, 1H, NH). ^13^C NMR (DMSO-*d*_6_, *δ* ppm): 15.21 (CH_3_), 44.18 (CH_2_), 50.76 (CH_2_), 51.51 (2CH_2_), 62.46 (CH_2_), arC: [108.93 (d, CH, *J*_C–F_ = 23.4 Hz), 113.47 (d, CH, *J*_C–F_ = 34.4 Hz), 117.88 (CH), 118.82 (C), 120.71 (CH), 121.51 (CH), 121.84 (CH), 122.84 (CH), 123.76 (d, CH, *J*_C–F_ = 41.0 Hz), 124.87 (C), 137.91 (d, C, *J* = 19.8 Hz), 139.24 (2C) 155.26 (d, C, *J*_C–F_ = 4.0 Hz)], 153.18 (N=CH), 185.74 (C=O).

##### Ethyl 4-(4-{[(benzylamino)carbonyl]amino}-2-fluorophenyl)piperazine-1-carboxylate (**5**)

The mixture of compound **3** (10 mmol) and benzylisothiocyanate (10 mmol) in absolute ethanol was refluxed for 10 h. On cooling the reaction mixture to room temperature, a solid formed. This crude product was collected by filtration and recrystallized from ethanol. Yield: 93 %. M.p: 153–155 °C. FT-IR (KBr, *ν*, cm^−1^): 3346, 3284 (2NH), 3063 (ar–CH), 1694, 1638 (2C=O), 1236 (C–O). Elemental analysis for C_21_H_25_FN_4_O_3_ calculated (%): C, 62.99, H, 6.29; N, 13.99. Found (%): C, 62.78; H, 6.07; N, 14.04. ^1^H NMR (DMSO-*d*_6_, *δ* ppm): 1.17 (t, 3H, CH_3_, *J* = 7.6 Hz), 2.85 (s, 4H, 2CH_2_), 3.40 (s, 4H, 2CH_2_ + H_2_O), 4.02 (q, 2H, CH_2_, *J* = 7.0 Hz), 4.26 (d, 2H, CH_2_, *J* = 6.0 Hz), 6.61 (brs, 1H, NH), 6.95 (s, 2H, arH), 7.21–7.31 (m, 6H, arH), 8.62 (s, 1H, NH). ^13^C NMR (DMSO-*d*_6_, *δ* ppm): 15.27 (CH_3_), 41.39 (CH_2_), 43.39 (CH_2_), 44.15 (CH_2_), 51.23 (CH_2_), 60.45 (CH_2_), 61.52 (CH_2_), arC: [106.69 (d, CH, *J*_C–F_ = 25.6 Hz), 114.19 (CH), 120.59 (CH), 127.42 (CH), 127.79 (2CH), 128.99 (2CH), 133.98 (d, C, *J*_C–F_ = 9.55 Hz), 137.02 (d, C, *J*_C–F_ = 9.85 Hz), 140.98 (C), 156.65 (d, C, *J*_C–F_ = 137.5 Hz)], 155.83 (2C=O).

##### Ethyl 4-(4-{[3-benzyl-4-oxo-1,3-oxazolidin-2-ylidene]amino}-2-fluorophenyl)piperazine-1-carboxylate (**6**)

The mixture of compound **5** (10 mmol) and ethyl bromoacetate in absolute ethanol was refluxed in the presence of dried sodium acetate (50 mmol) for 13 h. After removing the solvent under reduced pressure, a solid appeared. This crude product was washed water and the precipitated solid was recrystallized from ethanol:water (1:2). Yield: 64 %. M.p: 158–159 °C. FT-IR (KBr, *ν*, cm^−1^): 1696, 1638 (2C=O), 1429 (C=N), 1210 (C–O). Elemental analysis for C_23_H_25_FN_4_O_4_ calculated (%): C, 62.72; H, 5.72; N, 12.72. Found (%): C, 62.87; H, 5.98; N, 12.88. ^1^H NMR (DMSO-*d*_6_, *δ* ppm): 1.35 (t, 3H, CH_3_, *J* = 8.0 Hz), 3.02 (brs, 4H, 2CH_2_), 3.53 (s, 4H, 2CH_2_ + H_2_O), 3.65 (brs, 2H, CH_2_), 4.22 (q, 2H, CH_2_, *J* = 7.0 Hz), 4.44 (d, 2H, CH_2_, *J* = 5.8 Hz), 7.08–7.12 (m, 3H, arH), 7.43–7.49 (m, 5H, arH). ^13^C NMR (DMSO-*d*_6_, *δ* ppm): 15.26 (CH_3_), 43.37 (CH_2_), 44.16 (CH_2_), 51.24 (2CH_2_), 54.37 (CH_2_), 61.54 (CH_2_), 62.49 (CH_2_), arC: [105.9 (d, CH, *J*_C–F_ = 95.7 Hz), 114.21 (CH), 119.98 (d, CH, *J*_C–F_ = 61.1 Hz), 127.38 (CH), 127.78 (2CH), 128.97 (2CH), 133.72 (d, C, *J*_C–F_ = 30.1 Hz), 136.95 (d, C, *J*_C–F_ = 36.5 Hz), 142.15 (C), 143.15 (d, C, *J*_C–F_ = 211.6 Hz)], 155.30 (C=O), 155.92 (C=N), 161.28 (C=O). MS *m*/*z* (%): 479.16 ([M+K]^+^, 100).

##### 4-(4-{[3-Benzyl-5-(4-chlorophenyl)-1,3-oxazol-2(3*H*)-ylidene]amino}-2-fluorophenyl) piperazine-1-carboxylate (**7**)

The mixture of compound **5** (10 mmol) and 4-chlorophenacylbromide (10 mmol) in absolute ethanol was refluxed in the presence of dried sodium acetate (50 mmol) for 11 h. Then, the reaction mixture was cooled to room temperature and the precipitated salt was removed by filtration. After evaporating the solvent under reduced pressure, a solid appeared. This crude product recrystallized with ethyl acetate: petroleum ether (1:2). Yield: 40 %, M.p: 162–163 °C. FT-IR (KBr, *ν*, cm^−1^): 1697 (C=O), 1429 (C=N), 1209 (C–O). Elemental analysis for C_23_H_28_ClFN_4_O_3_ calculated (%): C, 65.10, H, 5.28; N, 10.47. Found (%): C, 65.14; H, 5.39; N, 10.49. ^1^H NMR (DMSO-*d*_6_, *δ* ppm): 1.17 (t, 3H, CH_3_, *J* = 7.6 Hz), 2.85 (s, 4H, 2CH_2_), 3.47 (s, 4H, 2CH_2_), 4.04 (q, 2H, CH_2_, *J* = 6.2 Hz), 4.26 (brs, 2H, CH_2_), 6.85–6.94 (m, 4H, arH + CH), 7.28 (brs, 8H, arH), 7.45 (s, 1H, arH). ^13^C NMR (DMSO-*d*_6_, *δ* ppm): 15.27 (CH_3_), 43.36 (2CH_2_), 44.14 (2CH_2_), 51.21 (CH_2_), 61.52 (CH_2_), 96.76 (CH), arC: [106.66 (d, CH, *J*_C–F_ = 25.6 Hz), 114.13 (CH), 120.50 (CH), 124.20 (2CH), 124.97 (2CH), 127.38 (CH), 127.78 (2CH), 128.97 (2CH), 133.90 (d, C, *J*_C–F_ = 21.9 Hz), 137.14 (d, C, *J*_C–F_ = 11.0 Hz), 141.05 (2C), 155.28 (C), 155.63 (d, C, *J*_C–F_ = 240.5 Hz)], 155.91 (C + C=O), 162.27 (C=N). MS *m*/*z* (%): 535.12 ([M]^+^, 14), 479.16 (100), 423.16 (97), 138.12 (50).

##### Ethyl 4-{4-[(2-ethoxy-2-oxoethyl)amino]-2-fluorophenyl}piperazine-1-carboxylate (**8**)

To the mixture of compound **3** (10 mmol) and triethylamine (10 mmol) in dry tetrahydrofurane, ethylbromoacetate (10 mmol) was added drop by drop at 0–5 °C. Then, the reaction mixture was allowed to reach room temperature and stirred for 14 h (the progress of the reaction was monitored by TLC). The precipitated triethylammonium salt was removed by filtration and the resulting solution was evaporated under reduced pressure to dryness. The obtained yellow solid was recrystallized from ethanol:water (1:2). Yield: 50.2 %. M.p: 71–73 °C. FT-IR (KBr, *ν*, cm^−1^): 3383 (NH), 1719 (C=O), 1697 (C=O), 1220 (C–O). Elemental analysis for C_17_H_24_FN_3_O_4_ calculated (%): C, 57.78; H, 6.85; N, 11.89. Found (%): C, 57.74; H, 6.77; N, 11.97. ^1^H NMR (DMSO-*d*_6_, *δ* ppm): 1.35 (t, 6H, 2CH_3_, *J* = 7.0 Hz), 2.95 (s, 4H, 2CH_2_), 3.60 (s, 6H, 3CH_2_), 4.24 (q, 4H, 2CH_2_, *J* = 7.0 *Hz*), 5.24 (s, 1H, NH), 6.44–6.59 (m, 2H, arH), 6.94–7.05 (m, 1H, arH). ^13^C NMR (DMSO-*d*_6_, *δ* ppm): 14.80 (CH_3_), 15.24 (CH_3_), 44.23 (CH_2_), 45.49 (2CH_2_), 51.33 (CH_2_), 51.75 (CH_2_), 61.01 (CH_2_), 61.52 (CH_2_), arC: [101.06 (d, CH, *J*_C–F_ = 24.1 Hz), 121.47 (d, CH, *J*_C–F_ = 4.0 Hz), 121.67 (d, CH, *J*_C–F_ = 4.0 Hz), 129.97 (d, C, *J*_C–F_ = 9.9 Hz), 145.96 (d, C, *J*_C–F_ = 10.6 Hz), 157.02 (d, C, *J*_C–F_ = 240.9 Hz)], 155.29 (C=O), 171.90 (C=O). MS *m*/*z*(%): 376.34 ([M+Na]^+^, 75), 354.38 ([M+1]^+^,100), 222.17 (22), 149.03 (49).

##### Ethyl 4-{2-fluoro-4-[(2-hydrazinyl-2-oxoethyl)amino]phenyl}piperazine-1-carboxylate (**9**)

Hydrazine hydrate (25 mmol) was added to the solution of compound **8** (10 mmol) in ethanol and the mixture was heated under reflux for 14 h. On cooling the mixture in cold overnight, a white solid appeared. The crude product was filtered off and recrystallized from ethyl acetate. Yield: 54 %. M.p: 153–155 °C. FT-IR (KBr, *ν*, cm^−1^): 3313 (2NH + NH_2_), 1675 (C=O), 1653 (C=O). Elemental analysis for C_15_H_22_FN_5_O_3_ calculated (%): C, 53.09; H, 6.53; N, 20.64. Found (%): C, 53.18; H, 6.79; N, 20.44. ^1^H NMR (DMSO-*d*_6_, *δ* ppm): 1.18 (t, 3H, CH_3_, *J* = 6.2 Hz), 2.77 (s, 4H, 2CH_2_), 3.37 (s, 4H, 2CH_2_), 4.05 (d, 2H, CH_2_, *J* = 7.0 Hz), 4.24 (s, 2H, CH_2_), 5.93 (brs, 2H, NH_2_), 6.25–6.39 (m, 2H, arH), 6.83 (t, 1H, arH, *J* = 9.8 Hz), 9.09 (s, 2H, 2NH). ^13^C NMR (DMSO-*d*_6_, *δ* ppm): 15.27 (CH_3_), 43.09 (CH_2_), 44.30 (CH_2_), 46.04 (CH_2_), 51.78 (2CH_2_), 61.48(CH_2_), arC: [101.10 (d, CH, *J* = 24.1 Hz), 108.53 (CH), 121.70 (CH), 130.00 (d, C, *J*_C–F_ = 9.5 Hz), 146.18 (d, C, *J*_C–F_ = 10.0 Hz), 157.03 (d, C, *J*_C–F_ = 240.9 Hz)], 155.26 (C=O), 169.97 (C=O). MS *m*/*z* (%): 380.47 ([M+2+K]^+^,100), 379.41 ([M+1 + K]^+^, 30), 267.22 ([M–CH_2_CONHNH_2_]^+^, 33), 234.18 (28).

##### Ethyl 4-(2-fluoro-4-{[2-(2-{[(4-fluorophenyl)amino]carbonothioyl}hydrazino)-2-oxoethyl]amino}phenyl)piperazine-1-carboxylate (**10**)

The solution of compound **9** (10 mmol) in absolute ethanol was refluxed with 4-fluorophenylisothiocyanate (10 mmol) for 10 h. On cooling the reaction mixture to room temperature, an oily product appeared. This was recrystallized from butyl acetate: ethyl ether (1:2). Yield: 50 %. M.p: 78–80 °C. FT-IR (KBr, *ν*, cm^−1^): 3225 (2NH + NH_2_), 1671 (2C=O), 1210 (C–O). Elemental analysis for C_22_H_26_F_2_N_6_O_3_S calculated (%): C, 53.66; H, 5.32; N, 17.06. Found (%): C, 53.78; H, 5.47; N, 17.14. ^1^H NMR (DMSO-*d*_6_, *δ* ppm): 1.19 (brs, 3H, CH_3_), 2.78 (s, 4H, 2CH_2_), 3.35 (s, 4H, 2CH_2_), 3.77 (brs, 2H, CH_2_), 4.06 (brs, 2H, CH_2_), 5.91 (brs, 2H, 2NH), 6.35 (brs, 2H, arH), 6.83 (brs, 1H, arH), 7.17 (brs, 2H, arH), 7.39 (brs, 2H, arH), 9.56 (brs, 1H, NH), 9.69 (brs, 1H, NH), 10.08 (brs, 1H, NH). ^13^C NMR (DMSO-*d*_6_, *δ* ppm): 17.45 (CH_3_), 43.56 (CH_2_), 46.49 (CH_2_), 53.96 (2CH_2_), 63.67 (CH_2_), 67.10 (CH_2_), arC: [105.40 (d, CH, *J*_C–F_ = 40.1 Hz), 114.19 (CH), 118.62 (d, CH, *J*_C–F_ = 36.6 Hz), 121.70 (2CH), 124.54 (2CH), 128.55 (d, C, *J*_C–F_ = 36.4 Hz), 140.19 (d, CH, *J*_C–F_ = 37.0 Hz), 150.36 (d, C, *J*_C–F_ = 184.7 Hz), 157.43 (2C)], 168.24 (C=O), 172.66 (C=O), 190.04 (C=S).

##### Ethyl 4-[4-({2-[2-(anilinocarbonothioyl)hydrazino]-2-oxoethyl}amino)-2-fluorophenyl] piperazine-1-carboxylate (**11**)

The mixture of compound **9** (10 mmol) and phenylisothiocyanate (10 mmol) in absolute ethanol was heated under reflux for 10 h. On cooling the reaction mixture to room temperature, a white solid appeared. This crude product was filtered off and recrystallized from ethanol. Yield: 85 %, M.p: 160–163 °C. FT-IR (KBr, *ν*, cm^−1^): 3340, 3256, 3193 (4NH), 1697 (C=O), 1633 (C=O), 1286 (C=S). Elemental analysis for C_22_H_27_FN_6_O_3_S calculated (%): C, 55.68; H, 5.73, N, 17.71. Found (%): C, 55.98; H, 5.78; N, 17.87. ^1^H NMR (DMSO-*d*_6_, *δ* ppm): 1.19 (t, 3H, CH_3_, *J* = 7.0 *Hz*), 2.78 (s, 4H, 2CH_2_), 3.47 (s, 4H, 2CH_2_), 3.77 (s, 2H, CH_2_), 4.04 (q, 2H, CH_2_, *J* = 7.2 Hz), 6.34–6.51 (m, 2H, arH), 6.80–6.85 (m, 1H, arH), 7.17 (s, 1H, arH), 7.34–7.38 (d, 4H, arH, *J* = 8.2 Hz), 9.56 (s, 1H, NH), 9.69 (s, 1H, NH), 10.12 (s, 2H, 2NH). ^13^C NMR (DMSO-*d*_6_, *δ* ppm): 15.29 (CH_3_), 44.25 (CH_2_), 45.92 (CH_2_), 51.83 (2CH_2_), 61.51 (2CH_2_), arC: [101.29 (d, CH, *J*_C–F_ = 24.1 Hz), 108.72 (CH), 121.68 (CH), 125.92 (2CH), 126.48 (CH), 128.82 (2CH), 139.70 (C), 146.20 (d, C, *J*_C–F_ = 10.0 Hz), 154.00 (d, C, *J*_C–F_ = 63.3 Hz), 157.35 (d, C, *J*_C–F_ = 209.8 Hz)], 168.64 (C=O), 170.64 (C=O), 181.58 (C=S). MS *m*/*z* (%): 475.41 ([M+1]^+^, 32), 414.53 (26), 413.53 (100), 149.03 (32).

##### Ethyl 4-(4-{[(5-anilino-1,3,4-thiadiazol-2-yl)methyl]amino}-2-fluorophenyl)piperazine-1-carboxylate (**12**)

Concentrated sulfuric acid (64 mmol) was added to compound **11** (10 mmol) dropwise while stirring, and the reaction mixture was stirred in an ice bath for 15 min. Then, the mixture was allowed to reach room temperature and stirred for additional 2 h. The resulting solution was poured into ice cold water and made alkaline (pH 8) with ammonia. The precipitated product was filtered, washed with water, and recrystallized from dimethysulfoxide:water (1:3). Yield 74 %. M.p: 93–95 °C. FT-IR (KBr, *ν*, cm^−1^): 3257 (2NH), 1677 (C=O), 1433 (C=N). Elemental analysis for C_22_H_25_F_2_N_6_O_2_S calculated (%): C, 57.88; H, 5.52; N, 18.41. Found (%): C, 58.08; H, 5.75; N, 18.78. ^1^H NMR (DMSO-*d*_6_, *δ* ppm): 1.17 (t, 3H, CH_3_, *J* = 7.0 Hz), 2.78 (s, 4H, 2CH_2_), 3.46 (s, 6H, 3CH_2_ + H_2_O), 4.03 (q, 2H, CH_2_, *J* = 7.2 *Hz*), 4.48 (s, 1H, NH), 6.37–6.51 (m, 2H, arH), 6.80–6.99 (m, 2H, arH), 7.17 (brs, 1H, NH), 7.27–7.33 (m, 3H, arH), 7.56 (d, 1H, arH, *J* = 7.8 Hz). ^13^C NMR (DMSO-*d*_6_, *δ* ppm): 14.47 (CH_3_), 42.36 (CH_2_), 43.37 (CH_2_), 45.14 (CH_2_), 50.03 (CH_2_), 50.92 (CH_2_), 60.72 (CH_2_), arC: [108.11 (d, CH, *J*_C–F_ = 12.4 Hz), 116.95 (d, CH, *J*_C–F_ = 19.4 Hz), 121.30 (d, CH, *J*_C–F_ = 33.3 Hz), 128.03 (CH), 128.75 (2CH), 128.96 (2CH), 129.53 (d, C, *J*_C–F_ = 9.5 Hz), 140.52 (C), 144.63 (d, C, *J*_C–F_ = 10.6 Hz), 156.51 (d, C, *J*_C–F_ = 204.2 Hz)], 160.77 (C), 164.32 (C), 169.87 (C=O). MS *m*/*z* (%): 458.16 ([M+2]^+^, 27), 457.16 ([M+1]^+^, 100).

##### Ethyl 4-[2-fluoro-4-({[4-(4-fluorophenyl)-5-thioxo-4,5-dihydro-1*H*-1,2,4-triazol-3-yl]methyl}amino)phenyl]piperazine-1-carboxylate (**13**)

A solution of compound **10** (10 mmol) in water was refluxed in the presence of 2 N NaOH for 3 h. Then, the resulting solution was cooled to room temperature and acidified to pH 4 with 37 % HCl. The precipitate formed was filtered off, washed with water, and recrystallized from ethanol. Yield: 61 %. M.p: 100–101 °C. FT-IR (KBr, *ν*, cm^−1^): 1675 (C=O), 1244 (C=S). ^1^H NMR (DMSO-*d*_6_, *δ* ppm): elemental analysis for C_22_H_24_F_2_N_6_O_2_S calculated (%): C, 55.68; H, 5.10; N, 17.71. Found (%): C, 55.54; H, 5.28; N, 17.89. ^1^H NMR (DMSO-*d*_6_, *δ* ppm): 1.18 (brs, 3H, CH_3_) 2.78 (brs, 4H, 2CH_2_), 3.41 (brs, 4H, 2CH_2_), 4.08 (brs, 4H, 2CH_2_), 5.87 (brs, 2H, 2NH), 6.27 (brs, 2H, arH), 6.79 (brs, 1H, arH), 7.45 (brs, 4H, arH). ^13^C NMR (DMSO-*d*_6_, *δ* ppm): 15.26 (CH_3_), 44.25 (2CH_2_), 51.67 (2CH_2_), 61.50 (2CH_2_), arC: [101.18 (d, CH, *J*_C–F_ = 9.5 Hz), 108.66 (CH), 116.98 (d, CH, *J*_C–F_ = 23.0 Hz), 121.53 (C), 130.31 (d, C, *J*_C–F_ = 10.2 Hz), 131.07 (2CH), 131.25 (2CH), 145.33 (d, C, *J*_C–F_ = 10.6 Hz), 153.16 (d, C, *J*_C–F_ = 213.8 Hz), 159.87 (d, C, *J*_C–F_ = 57.2 Hz)], 151.02 (C), 165.34 (C=O), 168.98 (C=S).

##### Ethyl 4-(2-fluoro-4-{[(4-phenyl-5-thioxo-4,5-dihydro-1*H*-1,2,4-triazol-3-yl)methyl] amino}phenyl)piperazine-1-carboxylate (**14**)

A solution of compound **11** (10 mmol) in ethanol water (1:1) was refluxed in the presence of 2 N NaOH for 3 h. Then, the resulting solution was cooled to room temperature and acidified to pH 7 with 37 % HCl. The precipitate formed was filtered off, washed with water, and recrystallized from ethyl acetate. Yield 70 %. M.p: 206–208 °C. FT-IR (KBr, *ν*, cm^−1^): 3248, 3117 (2NH), 3049 (ar CH), 1660 (C=O), 1250 (C=S). Elemental analysis for C_22_H_25_FN_6_O_2_S calculated (%): C, 57.88; H, 5.52; N, 18.41. Found (%): C, 57.51; H, 5.45; N, 18.49. ^1^H NMR (DMSO-*d*_6_, *δ* ppm): 1.13 (t, 3H, CH_3_, *J* = 7.4 Hz), 2.73 (s, 4H, 2CH_2_), 3.42 (s, 4H, 2CH_2_), 3.99 (s, 4H, 2CH_2_), 6.25–6.32 (m, 2H, arH + NH), 6.76–6.80 (m, 1H, arH), 7.36 (s, 2H, ar–H), 7.49 (brs, 4H, ar–H), 10.45 (s, 1H, NH). ^13^C NMR (DMSO-*d*_6_, *δ* ppm): 15.25 (CH_3_), 31.39 (CH_2_), 44.27 (2CH_2_), 51.68 (2CH_2_), 61.49 (CH_2_), arC: [101.27 (d, CH, *J*_C–F_ = 24 Hz), 108.63 (CH), 121.59 (CH), 128.76 (CH), 130.05 (2CH), 130.15 (2CH), 134.09 (2C), 145.50 (C), 150.92 (C)], 155.25 (C), 168.75 (C=S + C=O). MS *m*/*z* (%): 480.48 ([M+1 + Na]^+^, 29), 479.54 ([M+Na]^+^, 100), 457.41 ([M+1]^+^, 85).

##### ({[(6*R*,7*R*)-3-[(Acetyloxy)methyl]-7-({[3-[({4-[4-(ethoxycarbonyl)piperazin-1-yl]-3-fluorophenyl}amino)methyl]-4-(4-fluorophenyl)-5-thioxo-4,5-dihydro-1*H*-1,2,4-triazol-1-yl]methyl}amino)-8-oxo-5-thia-1-azabicyclo[4.2.0]oct-2-en-2-yl]carbonyl}oxy)triethyl ammonium (**15**)

7-Aca (10 mmol) was added to the mixture of compound **13** (10 mmol), triethylamine (20 mmol), and formaldehyde (50 mmol) in tetrahydrofurane, and the mixture was stirred at room temperature 4 h. After removing the solvent under reduced pressure, an oily product appeared. This was recrystallized from ethanol:water (1:2). Yield: 43 %. M.p: 68–70 °C. FT-IR (KBr, *ν*, cm^−1^): 3359, 3263 (2NH), 3075 (ar–CH), 2988, 2973 (aliphatic CH), 1680, 1629 (4C=O), 1228 (C=S). Elemental analysis for C_39_H_51_F_2_N_9_O7S_2_ calculated (%): C, 54.47; H, 5.98; N, 14.66. Found (%): C, 54.70; H, 5.74; N, 14.55. ^1^H NMR (DMSO-*d*_6_, *δ* ppm): 1.10 (brs, 12H, 4CH_3_) 1.74 (s, 3H, CH_3_), 2.86 (brs, 4H, 2CH_2_), 3.20 (s, 6H, 3CH_2_), 3.58 (brs, 6H, 3CH_2_), 4.04 (brs, 2H, CH_2_), 4.52 (brs, 2H, CH_2_), 4.67 (s, 4H, 2CH_2_), 4.89 (s, 2H, 2CH), 5.42 (s, 2H, 2NH), 6.51 (brs, 2H, arH), 6.89 (brs, 1H, arH), 7.35–7.44 (m, 4H, arH). ^13^C NMR (DMSO-*d*_6_, *δ* ppm): 9.01 (3CH_3_), 15.04 (CH_3_), 23.44 (CH_3_), 25.69 (CH_2_), 44.05 (2CH_2_), 46.25 (CH_2_), 49.16 (3CH_2_), 51.29 (CH_2_), 51.56 (2CH_2_), 54.70 (2CH), 61.89 (CH_2_), 67.78 (CH_2_), arC: [103.99 (d, CH, *J*_C–F_ = 12.45 Hz), 110.89 (CH), 117.08 (d, CH, *J*_C–F_ = 23.45 Hz), 120.97 (2CH), 131.04 (2CH), 131.69 (C), 131.88 (C), 143.85 (d, C, *J*_C–F_ = 9.85 Hz), 154.78 (d, C, *J*_C–F_ = 92.61 Hz), 162.96 (d, C, *J*_C–F_ = 246.0 Hz)], 130.41 (C), 130.49 (C), 150.18 (triazole-C), 165.79 (C=O), 168.64 (C=O), 168.86 (C=S), 171.93 (C=O), 175.76 (C=O).

##### [({(6*R*,7*R*)-3-[(Acetyloxy)methyl]-7-[({3-[({4-[4-(ethoxycarbonyl)piperazin-1-yl]-3-fluorophenyl}amino)methyl]-4-phenyl-5-thioxo-4,5-dihydro-1*H*-1,2,4-triazol-1-yl}methyl)amino]-8-oxo-5-thia-1-azabicyclo[4.2.0]oct-2-en-2-yl}carbonyl)oxy](triethyl)ammonium (**16**)

To the mixture of compound **14** (10 mmol), triethylamine (20 mmol) and formaldehyde (50 mmol) in tetrahydrofurane, 7-aca (10 mmol) was added. The mixture was stirred at room temperature 4 h. After removing the solvent under reduced pressure, an oily product appeared. This product recrystallized ethyl acetate:hexane (1:2). Yield: 47 %. M.p: 64–66 °C. FT-IR (KBr, *ν*, cm^−1^): 3662 (OH), 3374 (NH), 2988, 2901 (aliphatic CH), 1762 (C=O), 1687 (2C=O), 1629 (C=O), 1227 (C=S). Elemental analysis for C_39_H_52_FN_9_O_7_S_2_ calculated (%): C, 55.63; H, 6.22; N, 14.97. Found (%): C, 55.87; H, 6.33; N, 15.05. ^1^H NMR (DMSO-*d*_6_, *δ* ppm): 1.11 (t, 12H, 4CH_3_, *J* = 7.0 Hz), 1.99 (s, 3H, CH_3_), 2.99 (q, 8H, 4CH_2_, *J* = 8.0 Hz), 3.87 (brs, 10H, 5CH_2_), 4.55 (s, 2H, CH_2_), 4.68–4.80 (m, 4H, 2CH_2_), 5.40 (s, 2H, CH), 6.22 (brs, 2H, 2NH), 7.33 (brs, 3H, ar–H), 7.50–7.75 (m, 5H, ar–H).^13^C-NMR (DMSO-*d*_*6*_, *δ* ppm): 9.31 (3CH_3_), 15.22 (CH_3_), 21.38 (CH_3_), 25.79 (CH_2_), 41.30 (2CH_2_), 44.17 (2CH_2_), 45.79 (3CH_2_), 51.40 (CH_2_), 51.64 (CH_2_), 61.49 (CH_2_), 66.68 (CH_2_), 67.69 (CH), 71.09 (CH), arC: [110.41 (d, CH, *J*_C–F_ = 34.2 Hz), 118.31 (d, CH, *J*_C–F_ = 18.7 Hz), 123.22 (d, C, *J*_C–F_ = 22.1 Hz), 126.01 (CH), 128.74 (CH), 130.14 (2CH), 130.33 (2CH), 134.47 (d, C, *J*_C–F_ = 7.3 Hz), 148.01 (d, C, *J*_C–F_ = 172.2 Hz), 149.99 (C)], 138.25 (2C), 155.28 (C), 166.21 (C=S), 169.90 (C=O), 170.92 (C=O), 171.19 (C=O), 172.95 (C=O).

##### [({(5*R*,6*R*)-6-[({3-[({4-[4-(Ethoxycarbonyl)piperazin-1-yl]-3-fluorophenyl}amino)methyl]-4-phenyl-5-thioxo-4,5-dihydro-1*H*-1,2,4-triazol-1-yl}methyl)amino]-3,3-dimethyl-7-oxo-4-thia-1-azabicyclo[3.2.0]hept-2-yl}carbonyl)oxy](triethyl)ammonium (**17**)

To the mixture of compound **14** (10 mmol), triethylamine (20 mmol), and formaldehyde (50 mmol) in tetrahydrofurane, 6-apa (10 mmol) was added. The mixture was stirred at room temperature 4 h. After removing the solvent under reduced pressure, an oily product appeared. This product recrystallized ethyl acetate:hexane (1:2). Yield: 41 %, M.p: 64–66 °C. FT-IR (KBr, *ν*, cm^−1^): 3393 (NH), 3073 (ar–CH), 2980 (aliphatic CH), 1764 (C=O), 1692 (C=O), 1609 (C=O), 1230 (C–O). Elemental analysis for C_37_H_52_FN_9_O_7_S_2_ calculated (%): C, 56.54; H, 6.67; N, 16.04. Found (%): C, 56.65; H, 6.79; N, 16.87. ^1^H NMR (DMSO-*d*_6_, *δ* ppm): 1.13 (t, 12H, 4CH_3_, *J* = 6.2 Hz), 1.39 (brs, 3H, CH_3_), 1.42 (brs, 3H, CH_3_), 3.02 (q, 8H, 4CH_2_, *J* = 7.0 Hz), 3.43 (s, 8H, 4CH_2_), 3.73 (brs, 2H, CH_2_), 4.56 (s, 2H, 2CH), 5.41 (s, 2H, CH_2_), 6.24 (s, 1H, CH), 6.77 (brs, 1H, NH), 7.36 (brs, 3H, ar–H), 7.50 (s, 5H, ar–H). ^13^C-NMR (DMSO-*d*_6_, *δ* ppm): 8.99 (3CH_3_), 14.53 (CH_3_), 27.13 (2CH_3_), 43.49 (2CH_2_), 44.96 (2CH_2_), 50.58 (CH_2_), 50.70 (3CH_2_), 50.94 (2CH_2_), 60.75 (C-(CH_3_)_2_), 70.39 (CH), 73.89 (CH), 81.90 (CH), arC: [100.44 (d, CH, *J*_C–F_ = 24.1 Hz), 108.87 (d, CH, *J*_C–F_ = 213.1 Hz), 120.53 (d, CH, *J*_C–F_ = 60.2 Hz), 128.18 (CH), 129.57 (2CH), 129.64 (2CH), 133.79 (d, C, *J*_C–F_ = 14.9 Hz), 144.08 (d, C, *J*_C–F_ = 99.5 Hz), 146.84 (d, C, *J*_C–F_ = 442.1 Hz)] 149.26 (C), 154.53 (C), 156.88 (C=S), 167.90 (C=O), 168.09 (C=O), 170.16 (C=O).

##### Ethyl 4-[4-(3-{2-[5-(4-chlorophenyl)-3-phenyl-1,3-thiazol-2(3*H*)-ylidene]hydrazino}-3-oxoethyl)-2-fluorophenylamino]piperazine-1-carboxylate (**18**)

The mixture of compound **11** (10 mmol) and 4-chlorophenacylbromide (10 mmol) in absolute ethanol was refluxed in the presence of dried sodium acetate (50 mmol) for 12 h. After removing the solvent under reduced pressure, an orange solid appeared. This product washed water and recrystallized ethanol. Yield: 45 %. M.p: 60–62 °C. FT-IR (KBr, *ν*, cm^−1^): 3345, 3259 (2NH), 3054 (ar–CH), 1677 (C=O), 1628 (C=O). Elemental analysis for C_30_H_30_ClFN_6_O_3_S calculated (%): C, 59.15; H, 4.96; N, 13.80. Found (%): C, 59.05; H, 5.06; N, 13.87. ^1^H NMR (DMSO-*d*_6_, *δ* ppm): 1.15 (brs, 3H, CH_3_), 2.76 (s, 4H, 2CH_2_), 3.61 (s, 6H, 3CH_2_ + H_2_O), 4.03 (brs, 2H, CH_2_), 5.40 (s, 1H, NH), 6.44–6.54 (m, 1H, arH), 6.84–6.96 (m, 2H, arH + CH), 7.29–7.52 (m, 9H, arH), 7.95 (s, 1H, arH), 10.45 (s, 1H, NH). ^13^C NMR (DMSO-*d*_6_, *δ* ppm): 15.24 (CH_3_), 41.37 (CH_2_), 44.26 (CH_2_), 51.68 (CH_2_), 52.46 (2CH_2_), 61.48 (CH_2_), arC: [101.24 (d, CH, *J* = 24.5 Hz), 108.66 (CH), 117.54 (2CH), 120.12 (C), 121.75 (2CH), 122.41 (2CH), 128.76 (CH), 129.71 (2CH), 130.37 (2CH), 130.76 (2C), 131.82 (C), 139.37 (2C), 146.87 (d, C, *J*_C–F_ = 133.95 Hz)], 155.26 (C=N), 158.92 (C=O), 160.62 (C=O). MS *m*/*z* (%): 631.64 ([M−1 + Na]^+^, 25), 464.59 (26), 463.58 (83), 441.62 (26), 360.57 (61), 267.31 (29), 195.00 (40), 149.00 (100), 135.03 (50), 121.06 (65).

##### Ethyl 4-[2-fluoro-4-({2-[2-(3-hydroxy-4-methoxybenzylidene)hydrazino]-2-oxoethyl} amino)phenyl]piperazine-1-carboxylate (**19a**)

The mixture of solution of compound **9** (10 mmol) and 3-hydroxy-4-methoxybenzaldehyde (10 mmol) in absolute ethanol was irradiated by microwave at 200 W and 140 °C for 30 min. On cooling the reaction mixture to room temperature a solid was appeared. This crude product was recrystallized from ethanol. Yield: 72 %. M.p: 183–185 °C. FT-IR (KBr, *ν*, cm^−1^): 3342, 3181 (2NH), 3096 (ar–CH), 1678 (2C=O), 1437 (C=N), 1211 (C–O). Elemental analysis for C_23_H_28_FN_5_O_5_ calculated (%): C, 58.34; H, 5.96; N, 14.79. Found (%): C, 58.65; H, 6.06; N, 14.98. ^1^H NMR (DMSO-*d*_6_, *δ* ppm): 1.17 (t, 3H, CH_3_, *J* = 6.8 Hz), 2.77 (s, 4H, 2CH_2_), 3.36 (s, 6H, 3CH_2_), 3.78 (s, 3H, O–CH_3_), 3.99 (q, 2H, CH_2_, *J* = 6.6 Hz), 5.80 (brs, 1H, NH), 6.04 (brs, 1H, NH), 6.32–6.37 (m, 3H, arH), 6.84–6.98 (m, 3H, arH), 9.27 (s, 1H, N=CH), 11.35 (s, 1H, OH). ^13^C NMR (DMSO-*d*_6_, *δ* ppm): 15.26 (CH_3_), 44.29 (CH_2_), 44.62 (2CH_2_), 51.78 (2CH_2_), 56.22 (OCH_3_), 61.48 (CH_2_), arC: [101.23 (d, CH, *J*_C–F_ = 22.0 Hz), 108.47 (CH), 112.58 (d, CH, *J*_C–F_ = 15.0 Hz), 120.73 (CH), 120.96 (CH), 121.72 (CH), 127.64 (C), 129.83 (d, C, *J*_C–F_ = 9.1 Hz), 146.25 (C), 146.46 (C), 150.34 (d, C, *J*_C–F_ = 6.5 Hz), 151.36 (d, C, *J*_C–F_ = 388.7 Hz)], 144.44 (N=CH), 167.17 (C=O), 171.66 (C=O). MS *m*/*z* (%): 497.56 ([M+1 + Na]^+^, 31) 496.56 ([M+Na]^+^,100), 370.41 (19), 360.65 (22).

##### Ethyl 4-[2-fluoro-4-({2-oxo-2-[2-(pyridin-4-ylmethylene)hydrazino]ethyl}amino)phenyl] piperazine-1-carboxylate (**19b**)

The mixture of compound **9** (10 mmol) and pyridine-4-carbaldehyde (10 mmol) in absolute ethanol was irradiated by microwave at 200 W and 140 °C for 30 min. On cooling the reaction mixture to room temperature a solid was appeared. This crude product was recrystallized from ethanol. Yield: 85 %. M.p: 184–185 °C. FT-IR (KBr, *ν*, cm^−1^): 3356, 3269 (2NH), 3057 (ar–CH), 1707, 1679 (2C=O), 1428 (C=N), 1230 (C–O). Elemental analysis for C_21_H_25_FN_6_O_3_ calculated (%): C, 58.87; H, 5.88; N, 19.61. Found (%): C, 58.97; H, 6.00; N, 19.97. ^1^H NMR (DMSO-*d*_6_, *δ* ppm): 1.16 (brs, 3H, CH_3_), 2.76 (s, 4H, 2CH_2_), 3.41 (s, 4H, 2CH_2_), 4.02–4.03 (m, 2H, CH_2_), 4.21 (s, 2H, CH_2_), 6.35–6.51 (m, 2H, arH), 6.83 (brs, 1H, arH), 7.69 (brs, 2H, arH), 8.63 (s, 3H, 2arH + CH), 11.80 (s, 2H, 2NH). ^13^C NMR (DMSO-*d*_6_, *δ* ppm): 15.26 (CH_3_), 47.25 (CH_2_), 51.79 (2CH_2_), 52.85 (2CH_2_), 61.37 (CH_2_), arC: [107.70 (d, CH, *J*_C–F_ = 45.1 Hz), 114.07 (C), 118.26 (d, CH, *J*_C–F_ = 29.3 Hz), 120.15 (CH), 124.56 (2CH), 137.02 (C), 141.37 (d, C, *J*_C–F_ = 50.6 Hz), 146.20 (2CH), 152.26 (d, C, *J*_C–F_ = 161.2 Hz)], 150.31 (N=CH), 160.00 (C=O), 166.71 (C=O). MS *m*/*z* (%): 467.51 ([M+K]^+^, 19) 451.55 ([M+Na]^+^,65), 429.53 ([M+1]^+^, 76), 267.35 (45), 201.02 (23), 162.98 (25), 160.98 (30), 149.03 (100), 135.01 (72), 118.99 (71).

##### Ethyl 4-[2-fluoro-4-({2-[2-(2-hydroxybenzylidene)hydrazino]-2-oxoethyl}amino)phenyl] piperazine-1-carboxylate (**19c**)

The mixture of compound **9** (10 mmol) and 2-hydroxybenzaldehyde (10 mmol) in absolute ethanol was irradiated by microwave at 200 W and 140 °C for 30 min. On cooling the reaction mixture to room temperature a solid was appeared. This crude product was recrystallized from ethanol. Yield: 50 %. M.p: 155–157 °C. FT-IR (KBr, *ν*, cm^−1^): 3675 (OH), 3357, 3270 (2NH), 3059 (ar–CH), 1707, 1676 (2C=O), 1428 (C=N), 1230 (C–O). Elemental analysis for C_22_H_26_FN_5_O_4_ calculated (%): C, 59.58; H, 5.91; N, 15.79. Found (%): C, 59.72; H, 6.16; N, 15.77. ^1^H NMR (DMSO-*d*_6_, *δ* ppm): 1.17 (brs, 3H, CH_3_), 2.78 (s, 4H, 2CH_2_), 3.45 (s, 6H, 3CH_2_), 4.02–4.03 (m, 2H, CH_2_), 6.39 (brs, 2H, 2NH), 6.85 (brs, 4H, arH), 7.41 (brs, 3H, arH), 8.70 (s, 1H, N=CH), 10.56 (brs, 1H, OH). ^13^C NMR (DMSO-*d*_6_, *δ* ppm): 15.25 (CH_3_), 41.29 (CH_2_), 44.18 (2CH_2_), 51.51 (2CH_2_), 61.52 (CH_2_), arC: [108.24 (CH), 116.79 (d, CH, *J*_C–F_ = 36.2 Hz), 119.18 (C), 120.18 (CH), 122.19 (d, CH, *J*_C–F_ = 53.4 Hz), 126.61 (CH), 131.22 (CH), 132.68 (CH), 137.00 (C), 141.26 (d, C, *J*_C–F_ = 10.6 Hz), 152.71 (d, C, *J*_C–F_ = 252.9 Hz), 157.86 (C)], 146.15 (N=CH), 159.33 (C=O), 163.12 (C=O). MS *m*/*z* (%): 466.51 ([M+1+Na]^+^, 16), 444.55 ([M+1]^+^, 25), 249.20 (19), 241.19 (18), 149.03 (100), 135.07 (33), 121.06 (45), 103.04 (40).

##### Ethyl 4-(2-fluoro-4-{[(5-thioxo-4,5-dihydro-1,3,4-oxadiazol-2-yl)methyl]amino}phenyl) piperazine-1-carboxylate (**20**)

The mixture of compound **9** (10 mmol) and carbon disulfide (20 mmol) in absolute ethanol was refluxed in the presence of dried potassium hydroxide (10 mmol) for 13 h. Then, the resulting solution was cooled to room temperature and acidified with acetic acid. The precipitate formed was filtered off, washed with water, and recrystallized from ethyl acetate:petroleum ether (1:3) Yield 68 %. M.p: 210–212 °C. FT-IR (KBr, *ν*, cm^−1^): 3300 (2NH), 1675 (C=O), 1428 (C=N), 1249 (C=S). Elemental analysis for C_16_H_20_FN_5_O_3_S calculated (%): C, 50.38; H, 5.29; N, 18.36. Found (%): C, 50.51; H, 5.66; N, 18.74. ^1^H NMR (DMSO-*d*_6_, *δ* ppm): 1.17 (t, 3H, CH_3_, *J* = 6.6 Hz), 2.77 (s, 4H, 2CH_2_), 3.47 (s, 2H, CH_2_), 4.03 (q, 2H, CH_2_, *J* = 7.0 Hz), 4.34 (d, 2H, CH_2_, *J* = 5.0 Hz), 6.33–6.52 (m, 4H, ar-2H + 2NH), 6.85 (t, 1H, arH, *J* = 8.6 Hz). ^13^C NMR (DMSO-*d*_6_, *δ* ppm): 15.25 (CH_3_), 41.37 (2CH_2_), 44.25 (2CH_2_), 51.64 (CH_2_), 61.50 (CH_2_), arC: [101.41 (d, CH, *J*_C–F_ = 24.1 Hz), 108.78 (CH), 121.78 (CH), 130.67 (d, C, *J*_C–F_ = 9.9 Hz), 144.97 (d, C, *J*_C–F_ = 10.6 Hz), 156.95 (d, C, *J*_C–F_ = 241.9 Hz)], 155.28 (C=O), 163.00 (C), 185 (C=S).

##### ({[(6R,7R)-3-[(Acetyloxy)methyl]-7-({[5-[({4-[4-(ethoxycarbonyl)piperazin-1-yl]-3-fluorophenyl}amino)methyl]-2-thioxo-1,3,4-oxadiazol-3(2*H*)-yl]methyl}amino)-8-oxo-5-thia-1-azabicyclo[4.2.0]oct-2-en-2-yl]carbonyl}oxy)(triethyl)ammonium (**21**)

To the mixture of compound **20** (10 mmol), triethylamine (20 mmol) and formaldehyde (50 mmol) in tetrahydrofurane, 7-aca (10 mmol) was added. The mixture was stirred at room temperature 4 h. After removing the solvent under reduced pressure, a liquid product appeared. This was recrystallized by column chromatography (*n*-hexane:ethyl acetate, 4:1). Yield 58 %. FT-IR (KBr, *ν*, cm^−1^): 3373 (OH + NH), 2980, 2974 (aliphatic CH), 1676 (4C=O), 1432 (C=N), 1232 (C=S). Elemental analysis for C_33_H_47_FN_8_O_8_S_2_ calculated (%) C: 51.68; H: 6.18; N: 14.61. Found (%): C: 51.47; H: 6.00; N: 14.67. ^1^H-NMR (DMSO-*d*_*6*_) *δ* ppm: 1.12 (t, 12H, 4CH_3_, *J* = 7.0 Hz), 1.99 (s, 3H, CH_3_), 2.98–3.18 (m, 12H, 6CH_2_), 3.82 (brs, 8H, 4CH_2_), 4.00 (s, 2H, CH_2_), 4.56 (s, 2H, CH_2_), 4.65 (s, 1H, CH), 5.19 (s, 1H, CH), 6.40 (brs, 2H, 2NH), 6.90 (brs, 1H, ar–H), 6.94 (brs, 2H, ar–H). ^13^C-NMR (DMSO-*d*_*6*_) *δ* ppm: 9.33 (3CH_3_), 15.15 (CH_3_), 21.39 (CH_3_), 25.75 (CH_2_), 40.94 (CH_2_), 43.66 (CH_2_), 44.04 (CH_2_), 46.26 (2CH_2_), 48.64 (CH_2_), 50.95 (3CH_2_), 61.71 (CH_2_), 67.38 (CH_2_), 67.73 (CH), 70.89 (CH), arC: [107.63 (d, CH, *J*_C–F_ = 11.8 Hz), 113.45 (CH), 115.47 (CH), 120.42 (d, C, *J*_C–F_ = 34.7 Hz), 122.05 (C), 150.83 (d, C, *J*_C–F_ = 273.3 Hz)], 130.04 (C), 134.26 (C), 155.50 (C=O), 155.65 (C=O), 162.28 (C), 175.25 (2C=O), 189.74 (C=S).

##### ({[(5*R*,6*R*)-6-({[5-[({4-[4-(Ethoxycarbonyl)piperazin-1-yl]-3-fluorophenyl}amino)methyl]-2-thioxo-1,3,4-oxadiazol-3(2*H*)-yl]methyl}amino)-3,3-dimethyl-7-oxo-4-thia-1-aza bicyclo[3.2.0]hept-2-yl]carbonyl}oxy)(triethyl)ammonium (**22**)

To the mixture of compound **20** (10 mmol), triethylamine (20 mmol), and formaldehyde (50 mmol) in tetrahydrofurane, 6-apa (10 mmol) was added. The mixture was stirred at room temperature 6 h. After removing the solvent under reduced pressure, a liquid product appeared. This was recrystallized by column chromatography (*n*-hexane:ethyl acetate, 4:1). Yield 66 %. FT-IR (KBr, *ν*, cm^−1^): 3676 (OH), 2901, 2987 (aliphatic CH), 1768 (C=O), 1683 (2 C=O), 1431 (C=N), 1231 (C=S). Elemental analysis for C_31_H_47_FN_8_O_6_S_2_ calculated (%): C, 52.38; H, 6.66; N, 15.76. Found (%): C, 52.18; H, 6.79; N, 15.55. ^1^H-NMR (DMSO-*d*_*6*_, *δ* ppm): 0.99–1.21 (m, 18H, 6CH_3_), 2.90 (q, 8H, 4CH_2_, *J* = 7.0 Hz), 3.38 (q, 8H, 4CH_2_, *J* = 7.2 Hz), 3.98–4.08 (m, 4H, 2CH_2_), 4.55 (s, 1H, CH), 5.26 (s, 1H, CH), 5.30 (s, 1H, CH), 5.38, 5.45 (brs, 2H, 2NH), 6.80 (brs, 1H, ar–H), 6.94 (brs, 2H, ar–H). ^13^C-NMR (DMSO-*d*_*6*_, *δ* ppm): 9.32 (3CH_3_), 15.25 (CH_3_), 27.77 (CH_3_), 32.62 (CH_3_), 44.13 (CH_2_), 45.67 (2CH_2_), 51.09 (CH_2_), 51.50 (CH_2_), 52.61 (CH_2_), 56.73 (C–(CH_3_)_2_), 61.52 (CH_2_), 62.23 (CH_2_), 62.99 (CH_2_), 63.59 (CH_2_), 65.39 (CH), 67.00 (CH), 73.68 (CH), arC: [107.41 (d, CH, *J*_C–F_ = 9.8 Hz),113.72 (d, CH, *J*_C–F_ = 33.0 Hz), 120.07 (CH), 134,64 (d, C, *J*_C–F_ = 9.1 Hz), 143.12 (d, C, *J*_C–F_ = 9.5 Hz), 154.47 (d, C, *J*_C–F_ = 81.2 Hz)], 163.63 (C), 170.45 (C=O), 170.91 (C=O), 172.13 (C=O), 175.29 (C=S).

### Antimicrobial activity assessment

All bacterial and yeast strains were obtained from the Hifzissihha Institute of Refik Saydam (Ankara, Turkey) and were as follows: *Pseudomonas aeruginosa* ATCC 27853, *Enterococcus faecalis* ATCC 29212, *S. aureus* ATCC 25923, *B. cereus* 709 ROMA, Ms: *M. smegmatis* ATCC607, *C. albicans* ATCC 60193, Sc: *S. cerevisiae* RSKK 251. All the newly synthesized compounds were dissolved in dimethyl sulfoxide (DMSO) and ethanol to prepare chemicals of stock solution of 10 mg mL^−1^.

### Agar-well diffusion method

Simple susceptibility screening test using agar-well diffusion method as adapted earlier (Ahmad *et al*., 1998) was used. Each microorganism was suspended in Mueller–Hinton (MH) (Difco, Detroit, MI, USA) broth and diluted approximately to 106 colony forming unit (cfu) mL^−1^. They were “flood-inoculated” onto the surface of MH agar and Sabouraud dextrose agar (SDA) (Difco, Detriot, MI, USA) and then dried. For *C. albicans* and *C. tropicalis*, SDA were used. Five-millimeter diameter wells were cut from the agar using a sterile cork-borer, and 50 mL of the extract substances was delivered into the wells. The plates were incubated for 18 h at 35 °C. Antimicrobial activity was evaluated by measuring the zone of inhibition against the test organism. Ampicillin (10 mg) and Fluconazole (5 mg) were used as standard drugs. Dimethyl sulfoxide and ethanol were used as solvent controls. The antimicrobial activity results are summarized in Table 1.

**Table 1:** Screening for antimicrobial activity of the compounds (50 μL)

Comp. no	Microorganisms and inhibition zone (mm)								
	Ec	Yp	Pa	Sa	Ef	Bc	Ms	Ca	Sc
**2**	–	–	–	–	–	6	–	–	–
**3**	–	–	–	11	–	6	–	15	15
**4a**		8	8	–	–	–	10	8	8
**4b**	–	–	–	–	–	–	–	–	–
**4c**	–	–	–	–	–	–	–	8	8
**4d**	6	6	–	–	–	8	20	15	15
**4e**	–	–	–	–	–		20	10	10
**4f**	8	8	6	6	–	6	25	20	10
**5**	–	–	–	–	–	–	–	6	7
**6**	–	–	–	–	–	–	–	–	–
**7**	–	–	–	–	–	–	–	–	–
**8**	–	–	–	–	–	6	–	–	–
**9**	–	–	–	–	–	6	–	7	–
**10**	–	–	–	–	–	6	–	–	–
**11**	–	–	–	10	–	6	–	–	–
**12**	–	–	–	–	–	–	–	6	6
**13**	–	–	6	–	–	–	–	8	10
**14**	–	–	–	6	6	–	–	8	–
**15**	–	6	6	6	–	–	–	10	–
**16**	8	–	–	6	10	–	–	6	10
**17**	9	9	8	13	–	16	14	6	12
**18**	–	–	6	10	–	6	–	8	12
**19a**	–	–	6	–	8	–	–	9	6
**19b**	–	–	–	–	–	–	–	8	–
**19c**	–	–	6	–	8	–	–	8	6
**20**	–	–	–	10	6	6	15	8	12
**21**	8	8	–	6	10	10	20	10	8
**22**	9	8	15		9	10	18	8	12
Amp.	10	18	18	35	10	15			
Strep.							35		
Flu.								25	>25

(–), no activity

Ec, *Escherichia coli* ATCC 25922; Yp, *Yersinia pseudotuberculosis* ATCC 911; Pa, *Pseudomonas aeruginosa* ATCC 43288; Sa, *Staphylococcus aureus* ATCC 25923; Ef, *Enterococcus faecalis* ATCC 29212; Bc, *Bacillus cereus* 702 Roma; Ms, *M*. *smegmatis* ATCC607; Ca, *Candida albicans* ATCC 60193; Sc, *Saccharomyces cerevisiae* RSKK 251; Amp., Ampicillin; Strep., Streptomycin; Flu., Fluconazole

### Urease inhibition assay

Reaction mixtures comprising 25 μL of Jack bean urease, 55 μL of buffer (100 mM urea, 0.01 M K_2_HPO_4_, 1 mM EDTA, and 0.01 M LiCl, pH 8.2), and 100 mM urea were incubated with 5 μL of the test compounds at room temperature for 15 min in microtiter plates. The production of ammonia was measured by indophenol method and used to determine the urease inhibitory activity. The phenol reagent (45 μL, 1 % w/v phenol, and 0.005 % w/v sodium nitroprusside) and alkali reagent (70 μL, 0.5 % w/v sodium hydroxide, and 0.1 % v/v NaOCl) were added to each well and the increasing absorbance at 625 nm was measured after 20 min, using a microplate reader (Molecular Device, USA). The percentage inhibition was calculated from the formula 100 − (OD test well/OD control) × 100. Thiourea was used as the standard inhibitor. In order to calculate IC_50_ values, different concentrations of synthesized compounds and standard were assayed at the same reaction conditions (Weatherburn, 1967). The obtained results are presented in Table 2.

**Table 2:** Inhibitory activities of the synthesized compounds against Jack Bean urease

Compound	% Inhibition ± S.D.	IC_50_ ± S.D.
Thiourea	100 ± 0.1	54.56 ± 4.17
**2**	-^a^	–^b^
**3**	11 ± 3.3	–
**4a**	N.s.	–
**4b**	N.s.	–
**4d**	-	–
**4e**	1 ± 0.2	–
**4f**	-	–
**5**	-	–
**6**	3 ± 3.0	–
**7**	N.s.	–
**8**	7 ± 3.1	–
**9**	7 ± 3.0	–
**10**	4 ± 1	–
**12**	56 ± 4	–
**14**	-	–
**15**	100 ± 1.5	4.67 ± 0.53
**17**	100 ± 2.1	45.37 ± 0.78
**18**	-	–
**19a**	-	–
**19b**	47 ± 0.1	–
**19c**	-	–
**20**	N.s.	–

*N.s.* Not soluble

^a^No inhibition

^b^Not determined

### Anti-lipase activity assay

The inhibitory effects of those compounds were evaluated against porcine pancreatic lipase (PPL) (15 ng mL^−1^). Lipase activity assay was done according to Verger *et al*., (Woods *et al*., 2003). Microtiter plates were coated with purified tung oil TAGs. Compounds were mixed with PPL 1:2 (v/v) and incubated for 30 min. The microtiter plates containing purified tung oil, lipase solution, and assay buffer (10 mM Tris–HCl buffer, pH 8.0, containing 150 mM NaCl, 6 mM CaCl_2_, 1 mM EDTA, and 3 mg mL^−1^ *β*-cyclodextrin) were recorded continuously for 40 min against the buffer alone by using microplate reader (SpectraMax M5, Molecular Devices) at 272 nm. The inhibitory activity of those compounds and Orlistat, a positive control against pancreatic lipase, were measured at concentration of 6.25, 2.08, and 1.04 μg mL^−1^. Residual activities were calculated by comparing to control without inhibitor (T+). The assays were done in triplicate. The IC_50_ value was determined as the concentration of compound that give 50 % inhibition of maximal activity. The results are presented in Table 3.

**Table 3:** Porcine pancreatic lipase inhibitory activity of synthesized compounds

Compound no.	% Inhibition
**2**	–
**3**	–
**5**	–
**6**	16
**7**	33
**8**	22
**9**	20
**10**	–
**11**	–
**12**	68
**13**	63
**14**	75
**15**	73
**16**	6
**17**	–
**18**	1
**19a**	–
**19b**	–
**19c**	–
**20**	33
Orlistat	99
DMSO control	–
Positive control	–

All compounds were screened at concentration of 6.25 μg mL^−1^
